# Renal cell carcinoma escapes death by p53 depletion through transglutaminase 2-chaperoned autophagy

**DOI:** 10.1038/cddis.2016.14

**Published:** 2016-03-31

**Authors:** J H Kang, J-S Lee, D Hong, S-H Lee, N Kim, W-K Lee, T-W Sung, Y-D Gong, S-Y Kim

**Affiliations:** 1 Cancer Cell and Molecular Biology Branch, Research Institute, National Cancer Center, Goyang, Republic of Korea; 2 Cancer Immunology Branch, Division of Cancer Biology, Research Institute, National Cancer Center, Goyang, Republic of Korea; 3 Center for Innovative Drug Library Research, Dongguk University, Seoul, Korea; 4 Institute of Life Science and Natural Resources, Korea University, Seoul, Republic of Korea

## Abstract

In renal cell carcinoma, transglutaminase 2 (TGase 2) crosslinks p53 in autophagosomes, resulting in p53 depletion and the tumor's evasion of apoptosis. Inhibition of TGase 2 stabilizes p53 and induces tumor cells to enter apoptosis. This study explored the mechanism of TGase 2-dependent p53 degradation. We found that TGase 2 competes with human double minute 2 homolog (HDM2) for binding to p53; promotes autophagy-dependent p53 degradation in renal cell carcinoma (RCC) cell lines under starvation; and binds to p53 and p62 simultaneously without ubiquitin-dependent recognition of p62. The bound complex does not have crosslinking activity. A binding assay using a series of deletion mutants of p62, p53 and TGase 2 revealed that the PB1 (Phox and Bem1p-1) domain of p62 (residues 85–110) directly interacts with the *β*-barrel domains of TGase 2 (residues 592–687), whereas the HDM2-binding domain (transactivation domain, residues 15–26) of p53 interacts with the N terminus of TGase 2 (residues 1–139). In addition to the increase in p53 stability due to TGase 2 inhibition, the administration of a DNA-damaging anti-cancer drug such as doxorubicin-induced apoptosis in RCC cell lines and synergistically reduced tumor volume in a xenograft model. Combination therapy with a TGase 2 inhibitor and a DNA-damaging agent may represent an effective therapeutic approach for treating RCC.

Transglutaminase 2 (TGase 2, E.C. 2.1.2.13) is an enzyme that catalyzes an isopeptide bond between protein glutamine and lysine residues, resulting in a covalent crosslink.^1^ Previously, we found that, in renal cell carcinoma (RCC) cell lines, TGase 2 crosslinks p53 into aggregates in the autophagosome, resulting in p53 degradation by autophagy.^2^ This p53 instability allows tumor cells to evade apoptosis and grow. Instability of p53 in RCC is not associated with mutations because only 4% of samples of clear cell RCC in the COSMIC database present p53 mutations (Supplementary Figure 1). We recently reported that monotherapy using the TGase 2 inhibitor GK921 in a xenograft tumor model abrogated RCC growth through p53 stabilization.^3^ These results suggest the possibility of using TGase 2 inhibitors as a cancer therapy.^3^

p53 Regulation by human double minute 2 homolog (HDM2) involves proteasomal degradation through ubiquitination, whereas TGase 2-mediated p53 regulation involves autophagosome degradation. However, how TGase 2 depletes p53 in the context of the central regulation system of p53 under HDM2 surveillance is unknown. The half-life of p53 is very short due to its interaction with HDM2. HDM2 depletes p53 by binding to a region of its transactivation domain,^4^ which facilitates rapid turnover through ubiquitination.^5^ DNA damage-induced phosphorylation of the N terminus of p53 blocks its interaction with HDM2,^6^ which stabilizes p53 by inhibiting proteasome-mediated degradation.^7^

In this study, we found that silencing *TGM2*, the gene encoding TGase 2, in RCC cell lines induced cell death under starvation conditions through p53 stabilization to the same extent as did silencing of *HDM2*. This result suggests that the general instability of p53 in RCC largely depends on TGase 2-mediated autophagy. In fact, p53 depletion induced by TGase 2 has no energy cost to cancer cells because TGase 2 only uses calcium to catalyze covalent crosslinks. However, the role of TGase 2 in autophagy is not clearly understood in cancer, although it is known that TGase 2 knockout mice present impaired autophagy, which increases ubiquitinated protein aggregates upon starvation and deceases p62-dependent peroxisome degradation.^8^ The binding mechanism between TGase 2 and p62 is unknown, although TGase 2 is able to bind directly with p62, which often localizes in the autophagosome.^2, 8^ p62 (also known as sequestosome 1) is an adapter protein in the degradation of ubiquitinated proteins in autophagosomes through interaction with microtubule-associated protein 1 light chain 3 alpha (LC3).^9^ The N terminus of p62 is a PB1 (Phox and Bem1p-1) domain that binds to the PB1 region in the atypical protein kinase C, the MAPK kinase, and the NBR1 (neighbor of BRCA1 gene) protein.^10^ The C terminus of p62 harbors a ubiquitin-associated (UBA) domain^11^ that interacts with ubiquitin and polyubiquitin chains, and an LC3-interacting region domain that interacts with LC3 in phagophore membranes.^12^ The part of the p53–TGase 2 complex that binds to p62 is unknown, but could be a part of either p53 or TGase 2. We observed that p53 does not bind to p62 directly but is transferred to p62 through association with TGase 2.

Here, we explored the possibility that TGase 2 is a chaperone in autophagy by analyzing the domains of p53 and p62 that bind to TGase 2 as well as the domain of TGase 2 that binds to p62. Based on the finding that TGase 2 inhibition increases p53 stability, we also tested the possibility that DNA-damaging reagents such as doxorubicin expand the damage of p53-mediated cell death when combined with TGase 2 inhibition in a preclinical model of RCC.

## Results

### p53 is regulated competitively by TGase 2 and HDM2 in RCC under starvation

**Figure 1:**
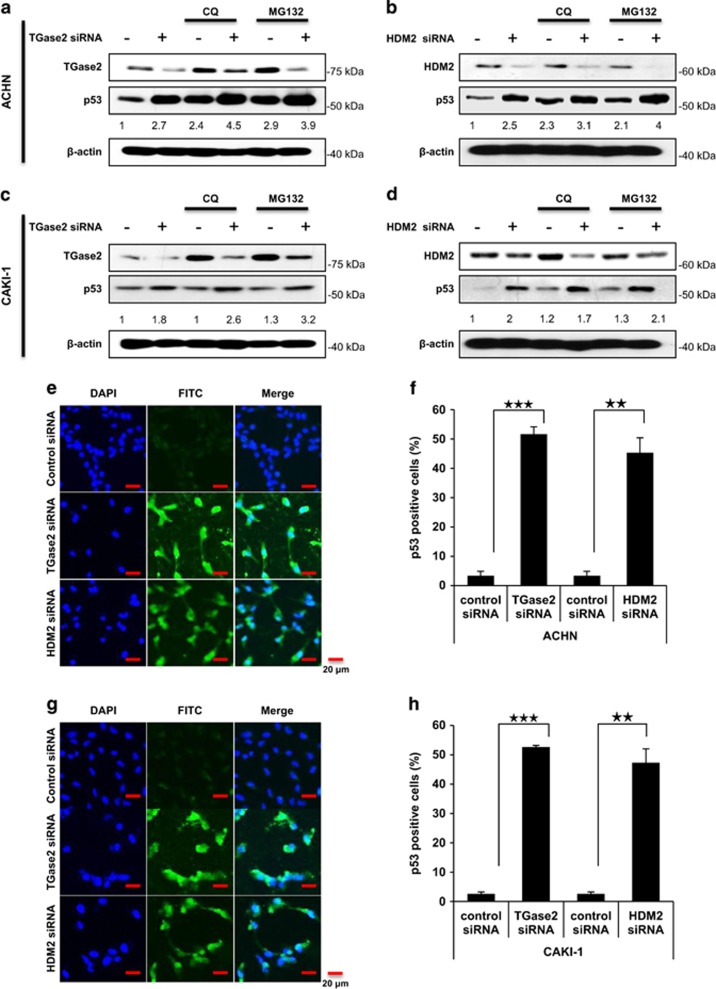
TGase 2 and HDM2 regulate p53 stability in an independent manner. ACHN (**a** and **b**) and CAKI-1 (**c** and **d**) cells were transfected with siRNA targeting *TGM2* (**a** and **c**) or *HDM2* (**b** and **d**) for 48 h; then the cells were treated with chloroquine (CQ, 50 *μ*M) or MG132 (10 *μ*M), or left untreated, for 6 h under starvation conditions before harvesting. Whole-cell lysates were subjected to immunoblotting with the indicated antibodies. ACHN (**e** and **f**) and CAKI-1 (**g** and **h**) cells were treated with siRNA for *TGM2* or *HDM2*, and stained with TUNEL and DAPI (**e** and **g**). Scale bar, 20 *μ*m. The percentage of TUNEL-positive cells was counted (**f** and **h**). The western blots are representative of three independent experiments. **P<0.01, ***P<0.001

To assess the relative contributions of TGase 2 and HDM2 in p53 depletion in RCC cell lines, the two corresponding genes were silenced using small interfering RNAs (siRNAs; Figure 1). The amount of p53 increased about 2.7-fold when *TGM2* was silenced, and about 2.5-fold with *HDM2* silencing in ACHN cells (Figures 1a and b). A similar pattern was observed in CAKI-1 cells, with a 1.8-fold and 2.0-fold increase, respectively (Figures 1c and d). This result suggests that p53 regulation depends equally on HDM2 and TGase 2 in RCC cells under starvation conditions.

We next examined whether the observed p53 stabilization was potentiated by treatment of chloroquine as an autophagy inhibitor or MG132 as a proteasome inhibitor. Treatment with chloroquine or MG132 alone stabilized p53 over twofold in both ACHN and CAKI-1 cells (Figures 1a–d). Combination treatment with both an siRNA and either chloroquine or MG132 obtained additional p53 stabilization (Figures 1a–d). Treatment with both siRNA for *TGM2* and chloroquine had the greatest effect on p53 stability, increasing its levels to 4.5-times the control level (Figure 1a), whereas the silencing of *HDM2* combined with MG132 increased p53 levels to four times the control level (Figure 1b). The apoptosis of ACHN and CAKI-1 cells to gene silencing was tested in a terminal deoxynucleotidyl transferase dUTP nick-end labeling (TUNEL) assay (Figures 1e–h). TUNEL showed that p53-positive cells increased in ACHN cells by about 16- and 14-fold in response to *TGM2* and *HDM2* silencing, respectively (Figures 1e and f). Similarly, in CAKI-1 cells, p53-positive cells increased by about 20- and 18-fold in response to *TGM2* and *HDM2* silencing, respectively (Figures 1g and h). Nutlin3a treatment onto RCC under normal culture media does not induce apoptosis that undergoes cell cycle arrest.^13^ However, Nutlin3a treatment under starvation induces remarkable apoptosis as we observed in HDM2 (Supplementary Figure 3).

### TGase 2 competes with HDM2 for binding to p53 in RCC

**Figure 2:**
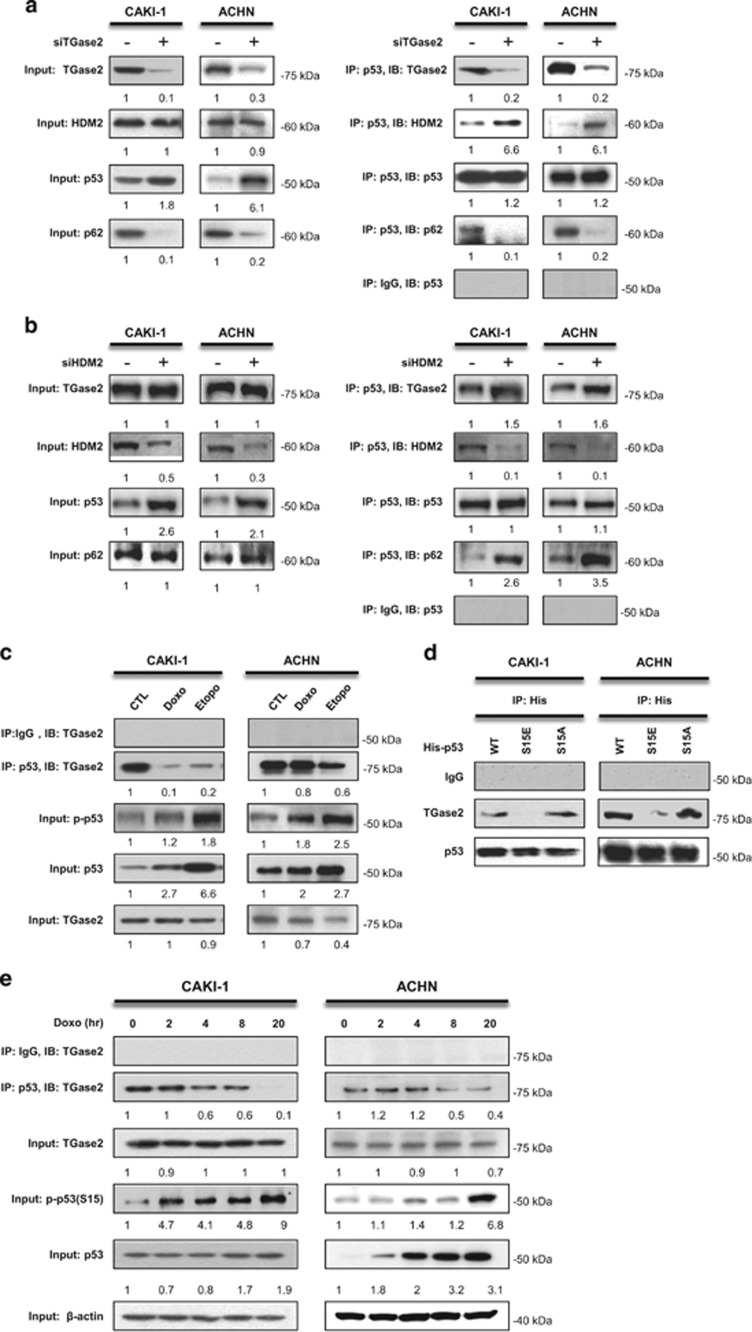
TGase 2 and HDM2 compete for p53 interaction. *TGM2* knockdown increased the interaction of p53 with HDM2, whereas it abolished the interaction with p62 (**a** and **b**). ACHN and CAKI-1 cells were transfected with siRNA for *TGM2* (**a**) or *HDM2* (**b**) for 48 h under starvation conditions. Whole-cell extracts (left) or p53 immunoprecipitates (right) were subjected to immunoblotting for TGase 2, HDM2, p53 and p62. (**c**) The induction of DNA damage inhibited the binding of TGase 2 to p53 and induced p53 phosphorylation. CAKI-1 and ACHN cells were treated with doxorubicin (1 *μ*M) or etoposide (50 *μ*M) for 24 h; whole-cell extracts were subjected to p53 immunoprecipitation and western blotting. (**d**) The activated p53 mimic (S15E) did not interact with TGase 2. CAKI-1 and ACHN were transfected with expression plasmids for p53 (wild type), p53 (S15E), or p53 (S15A) for 24 h, and the cell lysates were immunoprecipitated with a His-tag antibody and subjected to immunoblotting. (**e**) p53 stabilization (p-p53) by doxorubicin blocked interaction with TGase 2 in a time-dependent manner. After doxorubicin (1 *μ*M) treatment for up to 20 h, cell lysates were immunoprecipitated with an anti-p53 antibody and subjected to immunoblotting. The blots are representative of three independent experiments

To test whether TGase 2-dependent autophagic depletion of p53 is a collateral mechanism against HDM2-mediated p53 regulation, we used p53 immunoprecipitation to examine protein–protein binding (Figure 2). Silencing of *TGM2* increased the binding of HDM2 to p53 whereas it abolished the binding of p53 with p62 (Figure 2a). Knockdown of *HDM2* increased the binding of TGase 2 and p62 to p53 (Figure 2b). These results suggest that TGase 2 may bind to the same region of p53 where HDM2 binds, and that TGase 2 may chaperon p53 to p62.

Doxorubicin^14^ and etoposide^15^ induce rapid and extensive apoptosis through DNA damage and p53 phosphorylation that inhibits binding to HDM2. Therefore, we tested whether doxorubicin or etoposide treatment stabilizes p53 from TGase 2. Doxorubicin or etoposide treatment inhibited the binding of TGase 2 to p53, and induced p53 phosphorylation (Figure 2c). To identify whether this loss of function is directed to Ser15 phosphorylation of p53, a constitutively activated form of p53 (S15E) and an inactive form (S15A) were transfected in cells and tested for binding to TGase 2. The S15E mutant of p53 showed substantial loss of binding to TGase 2 (Figure 2d). TGase 2 lost its ability to bind p53 following induction of p53 phosphorylation at S15 in a time-dependent manner during doxorubicin treatment (Figure 2e). This result suggests that TGase 2 is a surveillance regulatory molecule against p53, like HDM2, due to the loss of binding activity when p53 is activated by DNA damage. However, participation of TGase 2 in p53 depletion through autophagy has an important role in the survival in RCC cells not only by reducing p53-dependent apoptosis, but also by supplying biomolecules through activation of autophagy under starvation conditions.

Previously, we identified TGase 2-targeting sites in p53 at multiple glutamines and lysines, including in the DNA-binding core (residues 102–292).^2^ In that study, we found that TGase 2 binds to the N terminus of p53 at the HDM2-binding domain (transactivating domain). This resembles HDM2 that binds to the N terminus of p53 and ubiquitinates multiple lysine residues including K370, 372, 373, 381, 382 and 386 in the C terminus of p53.^16^ We found that the TGase 2-binding domain and TGase 2-targeting sites in p53 are different because TGase 2 works as a chaperon of p53 to the autophagosome. This finding is in accordance with the observation that TGase 2-mediated crosslinking occurs only in the autophagosome during autophagy.^8^

### TGase 2 chaperones p53 to the autophagosome

**Figure 3:**
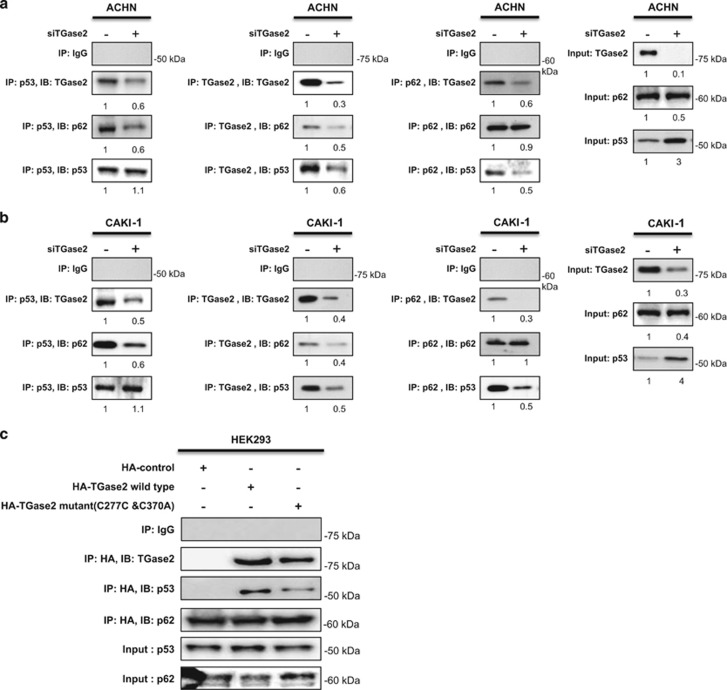
TGase 2 chaperones p53 to p62. (**a** and **b**) TGase 2 knockdown abolished the interaction of p53 to p62 as well as the interaction of TGase 2 to p53 and p62. *TGM2* was silenced in ACHN (**a**) or CAKI-1 (**b**) cells for 48 h under starvation conditions, and then cell extracts were subjected to immunoprecipitation of TGase 2, p53, and p62. (**c**) TGase 2 activity is not required for interacting with p53. Wild-type or catalytically inactive TGase 2 (double mutant, C277S and C370A) was co-transfected with p62 into HEK293 cells. TGase 2 was immunoprecipitated using an anti-HA-tag antibody, followed by immunoblotting of TGase 2, p53 and p62

TGase 2 transports p53 to the autophagosome for degradation by autophagy through polymerization. This idea was supported by immunoblotting of p53, which showed that high molecular weight polymers were only detected in the insoluble fraction after chloroquine treatment in RCC.^2^ Here, we tested the binding of three molecules, namely, TGase 2, p53, and p62, in ACHN (Figure 3a) and CAKI-1 (Figure 3b) cells. *TGM2* knockdown abolished p53 binding to p62 and significantly reduced p62 binding to p53. This result suggests that p53 does not bind to p62 directly and that TGase 2 is required for p53 autophagy in RCC. It is known that p62 is located in the autophagosome during autophagy. Therefore, this implies that p53 bound to TGase 2 transports to p62 by TGase 2–p62 binding. In other words, TGase 2 is a chaperone of p53 for autophagy.

Considering TGase 2 as a chaperone, its catalytic activity might not be necessary for chaperoning p53 in RCC. To test this possibility, an inactive, double mutant form of TGase 2 (C277S and C370A)^2, 17^ was transiently expressed in HEK293 cells, and cell extracts were subjected to immunoprecipitation using an anti-HA-tag antibody (Figure 3c). This mutant TGase 2 also bound p53 as well as p62 despite the lack of enzymatic activity.

### A series of deletion mutants reveals the interacting domains of p53 and p62

**Figure 4:**
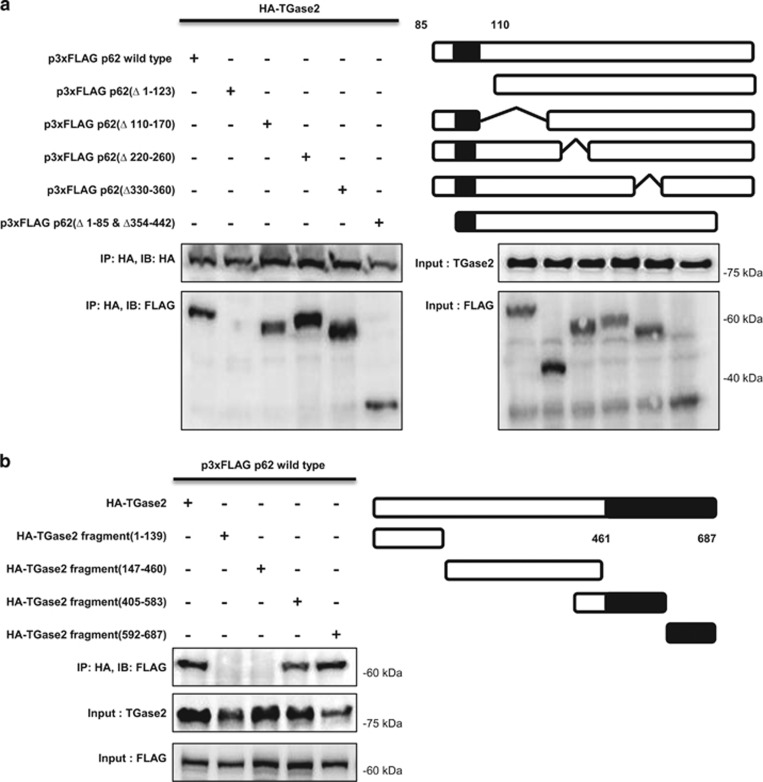

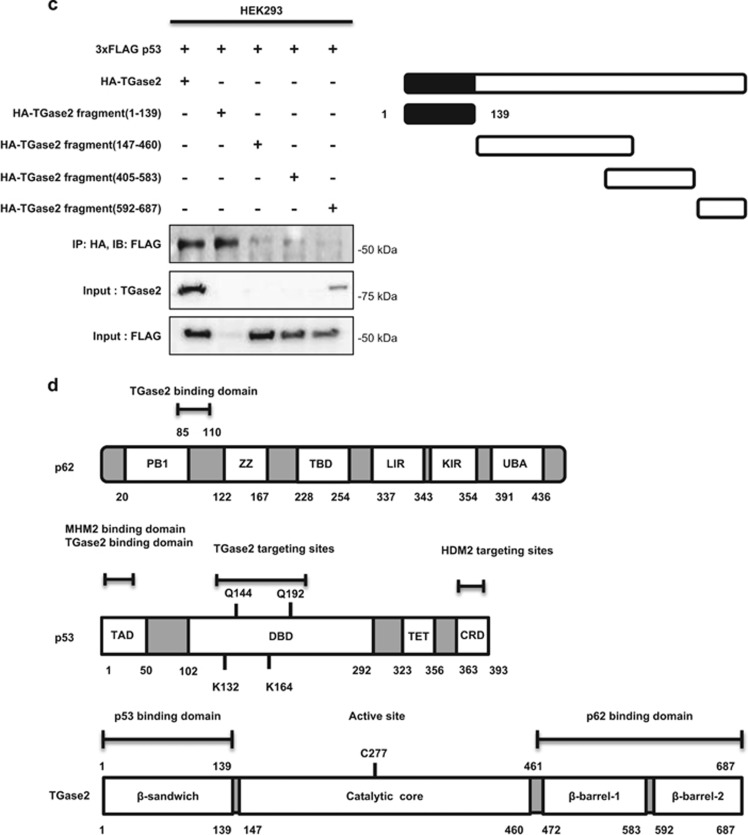
TGase 2 makes a trimer complex with p53 and p62. (**a**) The N terminus of p62 (residues 85–110) is the TGase 2-binding site. HEK293 cells were transfected with plasmids encoding HA-tagged TGase 2 (HA-TG2) and either wild-type p62 (p3xFLAG p62 wild type) or a p62 deletion mutant. The proteins were co-immunoprecipitated from cell extracts using an anti-HA-tag antibody and subjected to immunoblotting. (**b**) The C terminus of TGase 2 is the p62-binding domain (*β*-barrel domains, residues 461–687). HEK293 cells were transfected with p62 (p3XFLAG p62 wild type) and either wild-type TGase 2 (HA-TG2) or a TGase 2 deletion mutant. The proteins were co-immunoprecipitated from cell extracts using an anti-HA-tag antibody and subjected to immunoblotting. (**c**) The N terminus of TGase 2 is the p53-binding domain (residues 1–139). HEK293 cells were transfected with wild-type p53 (p3xFLAG p53) and either HA-tagged wild-type TGase 2 (HA-TG2) or a deletion mutant of TGase 2. Proteins were immunoprecipitated using an anti-HA-tag antibody. (**d**) Primary structures of p62, p53 and TGase 2 proteins. CRD, C-terminal regulatory domain, lysine rich domain; DBD, DNA-binding domain; KIR, Keap1-interacting region; LIR, LC3-interacting region; p53, tumor suppressor; p62, sequestosome 1; TAD, transcription-activation domain or transactivation domain; TBD, TRAF6-binding domain; TET, tetramerization domain; ZZ, zinc-finger domain

Next, to investigate which part of p62 binds to TGase 2, a series of FLAG-tagged deletion mutants of p62 was constructed, transfected into HEK293 cells together with HA-tagged TGase 2, and tested for binding ability by immunoprecipitation using an anti-HA-tag antibody. Deletion of the PB1 domain (residues 1–123) of p62 totally abolished binding to TGase 2 (Figure 4a). Four other deletion mutants retained binding, including one lacking residues 1–85 and 345–442. These results suggest that the region comprising residues 85–110 of p62 (a part of the PB1 domain) is responsible for interacting with TGase 2.

To determine which part of TGase 2 binds to this part of p62, a series of HA-tagged deletion mutants of TGase 2, designed in consideration of the protein's X-ray crystal structure,^17^ was tested by binding to full-length FLAG-tagged p62. Deletion recombinants missing the C terminus of TGase 2 had no p62-binding ability (Figure 4b). Therefore the C terminus region (residues 461–687) of TGase 2, which is composed of two *β*-barrel domains, has an important role in interacting with the PB1 domain of p62.

TGase 2 binds to p53 at its N terminus, which is also the HDM2-binding domain^18^ (Figure 2). To identify the p53-binding domain in TGase 2, a series of HA-tagged TGase 2 deletion recombinants was transiently expressed in HEK293 cells together with FLAG-tagged p53, and the proteins were immunoprecipitated from cell extracts with an anti-HA-tag antibody. A mutant TGase 2 consisting of the N terminus residues 1–139 retained binding ability to p53, whereas the constructs missing the N terminus did not bind p52 (Figure 4c).

The N terminus of TGase 2 (residues 1–139) is a fibronectin-binding domain^19^ that also interacts with the N terminus of p53, which also contains the HDM2-binding domain (residues 15–26). The TGase 2 C terminus, which consists of two *β*-barrel domains, is therefore available for interacting with the N terminus of p62 (residues 85–110), which consists of a PB1 domain. The C terminus of p62 is therefore free to interact with an autophagy molecule, LC3 (Figure 4d).

### TGase 2 inhibition combined with doxorubicin treatment potentiates cell death in RCC

**Figure 5:**
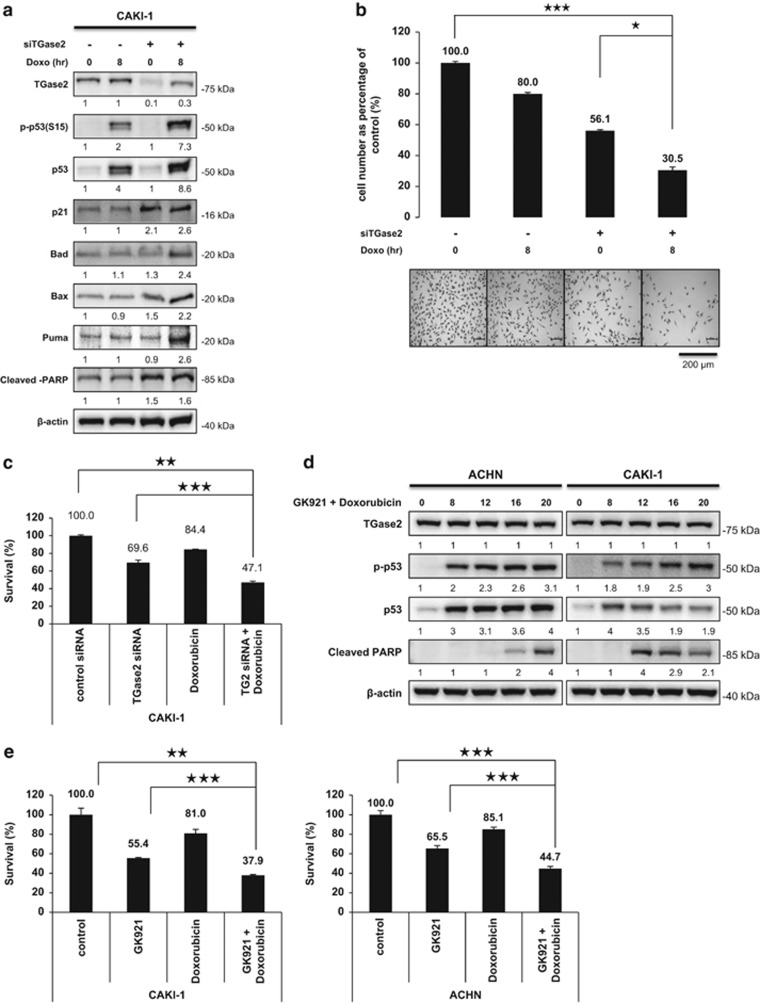
Doxorubicin potentiates the apoptotic effects of TGase 2 silencing by extending p53 stability. (**a**) CAKI-1 cells were treated with siTGase 2 for 48 h, and then with doxorubicin for an additional 8 h before harvesting for immunoblotting of TGase 2, p53 and downstream markers of p53. (**b**) Viable cell was counted by using SRB staining after a single treatment of control siRNA, siTGase 2, or doxorubicin (1 *μ*M), or a combined treatment of doxorubicin (1 *μ*M) and siTGase 2. *Y*-axis presents viable cell per square mm. (**c**) To measure combination effect of doxorubicin with TGase 2 knockdown against RCC growth, XTT assay was employed under same conditions as (**b**). (**d**) To measure induction of apoptosis by dual treatments, CAKI-1 and ACHN cells were treated with a combination of GK921 and doxorubicin (1 *μ*M) for 0, 8, 12, 18 and 20 h. Immunoblotting was performed against cleaved PARP and p-p53. (**e**) To measure combination effect of doxorubicin and GK921 against RCC growth, XTT assay was employed under same conditions as (**d**). Cumulative data from three independent experiments is shown here as mean±S.D. (*n*=3). *P<0.05, **P<0.01, ***P<0.001

Doxorubicin induces apoptosis by p53 stabilization through DNA damage. Previously, we found that TGase 2 inhibition induces cell death by p53 stabilization.^2^ Therefore, RCC may evade doxorubicin-induced cell death if the stabilized p53 is depleted by a TGase 2-mediated autophagy process through transportation to p62. To test this possibility, CAKI-1 cells were simultaneously treated with siTGase 2 and doxorubicin (Figure 5a). The combination increased p53 stability and led to cell death by activating p53 downstream markers including p21, BAD, BAX and PUMA (Figure 5a). However, p53 knocked down in CAKI-1 cell did not induce apoptosis by TGase 2 inhibition using GK921 (Supplementary Figure 2d). Cell survival assay using SRB staining showed that a single treatment of siRNA of TGase 2 or of doxorubicin reduced cell number by about 50 or 20% respectively, whereas the combination of siRNA of TGase 2 and doxorubicin reduced cell number by about 70% (Figure 5b). To measure combination effect of doxorubicin with TGase 2 knockdown against RCC growth, XTT assay was employed under same conditions as above. XTT assay showed that a single treatment of siRNA of TGase 2 or of doxorubicin reduced cell number by about 30 or 15% respectively, whereas the combination of siRNA of TGase 2 and doxorubicin reduced cell number by about 53% (Figure 5c).

To measure induction of apoptosis by dual treatments, CAKI-1 and ACHN cells were treated with a combination of GK921 and doxorubicin for 0, 8, 12, 18 and 20 h. Immunoblotting showed that increase of both p53 and p-p53 led to induction of PARP cleavage in 12 h (Figure 5d). To measure combination effect of doxorubicin and GK921 against RCC growth, XTT assay was employed under same conditions as above. XTT assay showed that a single treatment of GK921 or of doxorubicin reduced cell number by about 45 or 20% respectively, whereas the combination of GK921 and doxorubicin reduced cell number by about 70% (Figure 5e).

### Combination treatment with GK921 and doxorubicin obliterates RCC *in vivo*

**Figure 6:**
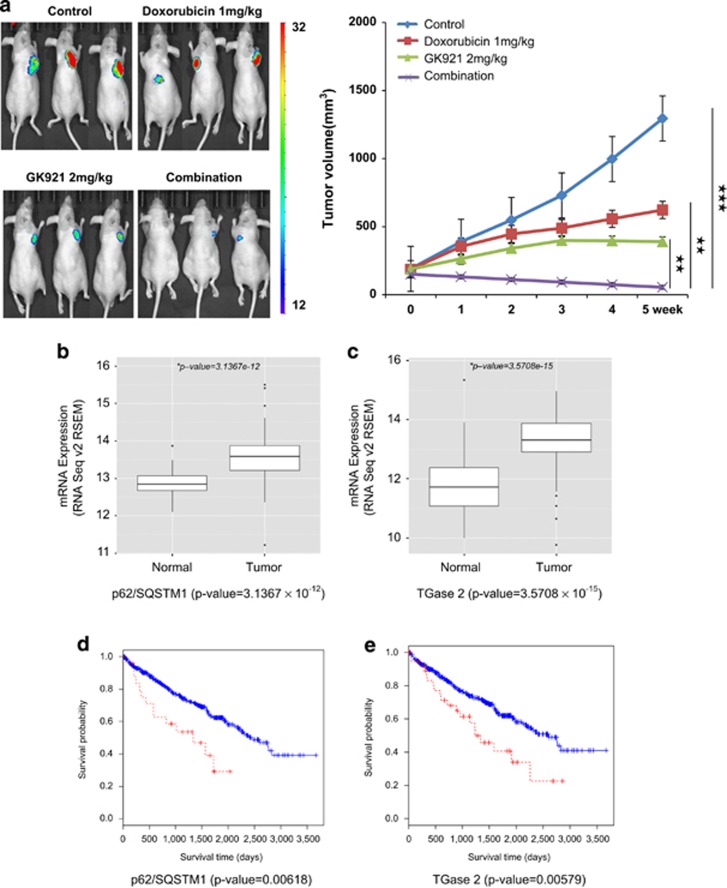
Doxorubicin amplifies the therapeutic response of RCC to the TGase 2 inhibitor GK921. (**a**) ACHN-luciferase cells were injected subcutaneously into 6–8-week-old female BALB/c nude mice. When the tumor volume reached 100 mm^3^, mice were treated orally, 5 days/week, with GK921 (2 mg/kg/day), doxorubicin (1 mg/kg/day), both or vehicle (*n*=3 per each group). After 5 weeks of treatment, tumor size was measured by the photon flux from luciferin and expressed on a color scale and as photons/s/cm^2^/steradian (left). Tumor volume was also measured using calipers (right). (**b**–**e**) Bioinformatics analysis of p62 gene (*SQSTM1*) and TGase 2 gene (*TGM2*) expression in clear cell RCC patients. (**b**) Lower p62/*SQSTM1* expression in normal kidney samples than in matched clear cell RCC samples (*P*=3.1367 × 10^−12^). (**c**) Lower *TGM2* expression in normal kidney samples than in matched clear cell RCC samples (*P*=3.5708 × 10^−15^). (**d**) Patients with high levels of p62/*SQSTM1* expression (red) had lower overall survival (OS) than those with low levels (blue). Log-rank test, *P*=0.00618. (**e**) Patients with high levels of expression of *TGM2* (red) also had a reduced OS. Gene expressions are colored red; higher levels of gene expression cut off *z*-score>2*σ*. Log-rank test, *P*=0.00579

We tested whether a therapy combining the TGase 2 inhibitor GK921 (ref. 3) with doxorubicin had a synergistic effect on a mouse xenograft model of RCC (Figure 6). Female BALB/c nude mice received ACHN cells subcutaneously and then were treated orally with the two drugs alone or in combination. No physical toxicity due to the drugs was observed in mice treated with combination of GK921 and doxorubicin for 2 weeks (data not shown). Oral administration of GK921 (2 mg/kg/day) and doxorubicin (1 mg/kg/day) began when tumors reached a volume of 100 mm^3^ and proceeded for 5 days per week. After 5 weeks of treatment, tumor volumes were significantly different between GK921-treated mice and vehicle-treated controls, and in mice that received the combined treatment, tumor volumes had receded substantially (Figure 6a). Others reported that combination treatment of 5-FU (8 mg/kg/day) and sorafenib (15 mg/kg/day) using CAKI-1 cells showed about 60% remission of tumor volume at day 42 while single treatment of sorafenib resulted in 40% remission.^20^ This combination result is remarkable therapeutic effect because tumor volume is decreased below the initial tumor volume without any side effect of drugs.

The mRNA expression levels of p62/*SQSTM1* and *TGM2* in clear cell RCC patients included in TCGA were remarkably and significantly higher than those in matched normal kidney tissues (Figures 6b and c). In this data set, moreover, ~5% and ~9% of cases (26/504 and 47/504) exhibited higher than average levels of expression of p62/*SQSTM1* and *TGM2*. A survival analysis demonstrated that patients with higher than average expression levels of p62/*SQSTM1* and *TGM2* had worse overall survival (Figures 6d and e). In our previous study,^2^ *TGM2* upregulation in clear cell RCC patients suggested a potential clinical therapeutic target. Based on this bioinformatics analysis, we suggest that TGase 2-mediated autophagy promotes tumor cell survival through chaperoning p53 to p62.

### A proposed mechanism of TGase 2-mediated chaperoning of p53 to the autophagosome

**Figure 7:**
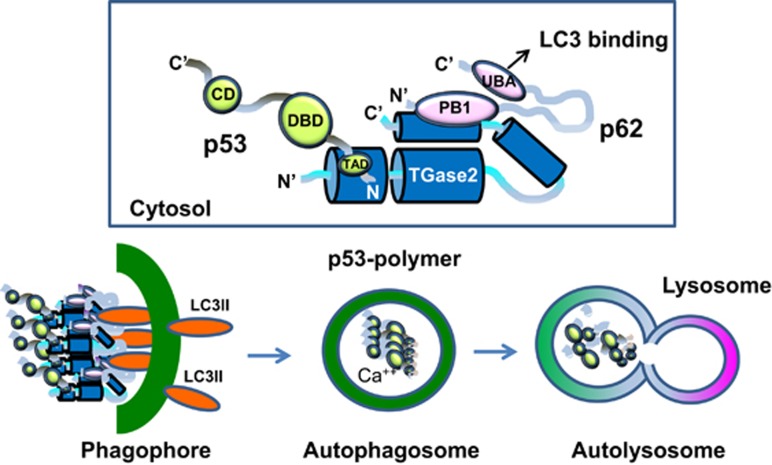
Model of the interactions of TGase 2, p53 and p62 based on the experimental data. The N terminus of TGase 2 interacts with the N terminus of p53, and the C terminus of TGase 2 interacts with the N terminus of p62 simultaneously. The C terminus of p62 in the complex is free and transfers the complex to LC3 in the phagophore. P53 is polymerized in the autophagosome when calcium increases. Later, the autophagosome and lysosome fuse into an autolysosome, which degrades all crosslinked materials. The TGase 2-binding site of p53 was identified as the HDM2-binding site in Figures 2d and e. Previously, we found that the TGase 2-targeting site is the DBD domain. CD, complexing domain; DBD, DNA-binding domain

Structural modeling of the triple complex of p53–TGase 2–p62 suggests that TGase 2 is a good chaperone molecule for p53 transportation to p62 without catalytic activity (Figure 7). This model is supported by our experiment in which an inactive form of TGase 2 bound p53 and p62 (Figure 3c). TGase 2 in the autophagosome is activated by an increase in calcium concentration, which crosslinks p53 monomers into polymers. These protein aggregates are then degraded by lysosomal proteolytic enzymes when the autophagosome fuses with the lysosome (Figure 7).

## Discussion

RCC survives and grows by inactivating p53 through TGase 2-mediated autophagy, which supplies recycled biomolecules under conditions of starvation. RCC patients with high *TGM2* expression had poor overall survival. It is not surprising that p53 levels are suppressed in RCC, although only 4% of RCC samples have p53 mutations. Therefore, TGase 2 is a possible therapeutic target for treating RCC, and this has been proved in a xenograft model.^3^ We found that DNA-damaging reagents such as doxorubicin enhance the p53 stability provided by the TGase 2 inhibitor GK921. We previously reported that monotherapy with the TGase 2 inhibitor GK921 had an anti-cancer effect under 8 mg/kg/day in an RCC xenograft model. In this study, combination therapy with doxorubicin (1 mg/kg/day) and GK921 (2 mg/kg/day) caused the RCC tumor to recede below the initial tumor volume. Therefore, combination therapy with these two agents represents a promising therapeutic approach to RCC.

In this study, we revealed the mechanism by which TGase 2 transports p53 to the autophagosome. We observed that the N terminus of TGase 2 binds the N terminus of p53 regardless of mutations and simultaneously associates with the N terminus of p62 through the C terminus of TGase 2. The LC3-binding domain of p62 is located in the C terminus of p62, which allows the formation of autophagosomes, whereas p62 interacts with a complex of TGase 2 and p53. During translocation of p53 to the autophagosome through TGase 2 binding, crosslinking activity is not needed. This finding is in agreement with the observation that TGase 2 crosslinking activity occurs only in the autophagosome during autophagy.^8^ This suggests that TGase 2 acts as a chaperone of p53 with a crosslinking catalytic activity. This interaction may result in rapid autophagy without consuming energy to tag ubiquitin on p53, as p62 is known to interact with ubiquitinated proteins. This autophagy process is beneficial in that it supplies building blocks, including degraded p53, for cancer cells. The N terminus of TGase 2 interacts with the N terminus of p53 and, simultaneously, the C terminus of TGase 2 interacts with the N terminus of p62; as a result, a heterotrimeric complex (p53–TGase 2–p62) is formed. The C terminus of p62, in the p53–TGase 2–p62 complex, is free and moves the complex to LC3 in the phagophore. When the autophagosome is completed with components of the phagophore, p53 is polymerized by TGase 2 with calcium-dependent activation in the autophagosome. Later, the autophagosome and lysosome are fused into an autolysosome, which degrades all crosslinked materials.

P62 has multiple physiological roles in cancer.^21^ The PB1 domain of p62 inhibits ERK1, which is a critical regulator of adipogenesis. Therefore, TGase 2 binding to the PB1 domain of p62 may increase ERK1 activity. Polyubiquitination consumes a lot of ATP to produce polyubiquitinated proteins, which are recognized by the UBA domain of p62 for autophagy. However, TGase 2-mediated p53 chaperoning to p62 is ubiquitination-independent through binding to the PB1 domain. The negatively charged, unfolded N terminus of p53, apart from the next α-helix (residues 15–29), may interact with the positively charged region of the N terminus of TGase 2 (residues 28–35).

Although the median progression-free survival has been increased by targeting tumor angiogenesis,^22^ about one-third of RCC patients develop metastatic disease.^23^ The main reason for metastatic disease is resistance to chemotherapy. Cancer cells are able to survive through autophagy, which is induced by many targeted kinase inhibitors.^24, 25^ It is well known that treatment with the mTOR inhibitor rapamycin induces autophagy by releasing mTOR mediated by suppression of autophagy. Therefore, autophagy can be induced not only by a lack of nutrient supply due to blood vessel shortage but also by drug treatment. A recent preclinical study reported that an mTOR 1/2 dual inhibitor combined with the autophagy inhibitor 3-methyaldenine resulted in a good outcome in an RCC model.^26^ Under drug-induced autophagy conditions, it remains to be determined whether TGase 2 is induced for p53 depletion through autophagy.

When RCC is targeted by angiogenesis with sorafenib, autophagy is also induced and correlates with drug resistance.^27^ In this study, we found that TGase 2 induces p53 depletion through transportation to p62 in RCC. Therefore, RCC can evade p53-dependent apoptosis by TGase 2 expression, which is induced by treatment with an anti-cancer drug such as doxorubicin.^28^ RCC may take advantage of the fact that TGase 2 destabilizes p53 activation induced by doxorubicin. Indeed, RCC evades cell death through TGase 2-mediated p53 depletion, which correlates with overall survival of RCC patients. Doxorubicin treatment combined with TGase 2 inhibition with GK921 inhibited RCC growth in a xenograft model even though the dose of GK921 used was below that needed for efficacy in monotherapy. Therefore, this combination therapy may represent a new approach to treating drug-resistant RCC.

## Materials and Methods

### Antibodies

The TGase 2 antibody from Lab Vision (clone CUB 7402; Fremont, CA, USA), the *β*-actin (SC-47778), HDM2 (SC-813), His (SC-803) and p53 (SC-126) and p62/SQSTM1 (SC-SC-25575), HA (SC-7392, SC-805) antibody from Santa Cruz Biotechnology (Santa Cruz, CA, USA), and p-p53(ser15) (#9284 S) LC3B (#3868) antibodies from Cell Signaling Technologies (Beverly, MA, USA) and FLAG(F1804, F7425) antibody from Sigma-Aldrich (St. Louis, MI, USA). We obtained Lipofectamine 3000, Lipofectamine RNAiMAX transfection regents, and negative control siRNA from Invitrogen (Carlsbad, CA, USA).

### Cell culture and treatments

Cell lines including ACHN, CAKI-1 and HEK293 were obtained from American Type Culture Collection (CRL1611, HTB-46, CRL1573 respectively; ATCC, Manassas, VA, USA). ACHN-luciferase cell line was obtained from Caliper Life Sciences (125056, Hopkinton, MA, USA). Cells were cultured in RPMI 1640 containing 10% fetal bovine serum under 5% CO_2_ and 100% humidity at 37 °C. A siRNA duplex targeting human TGM2 (siTGase 2; #10620318, #10620319, Invitrogen) and HDM2 was treated to cells for 48 h using Lipofectamine RNAiMAX (Invitrogen, Carlsbad, CA, USA), according to the manufacturer's instructions. As negative controls, cells were incubated with Lipofectamine RNAiMAX (Invitrogen) and a negative siRNA (Santa Cruz). Then, cells were left untreated or were treated with 50 *μ*M of chloroquine to inhibit autophagolysosome fusion or 10 *μ*M of MG132 to prevent proteasome degradation in amino acid-free Earle's balanced salts solution (EBSS; Sigma-Aldrich) for 6 h. In this study, siRNA was the mix of three individual siRNA duplexes per target gene designed using Rosetta Inpharmatics′ algorithm from company. As reviewer suggested a proof of the same effect using at least two independent siRNA, we have repeated experiments with two different siRNA sequences against targets of TGase 2, HDM2 and p53, which resulted in the same effect as the mix of three siRNAs (Supplementary Figure 2).

### Plasmid construct


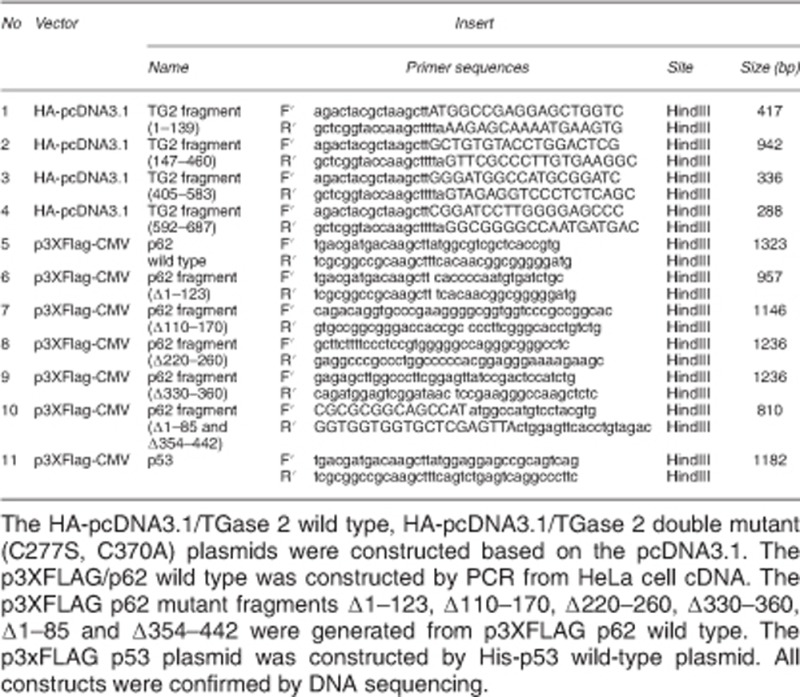


### Western blotting and immunoprecipitation

For western blot analysis, cells were lysed with radio-immunoprecipitation assay (RIPA) buffer and the protein concentration of the lysates was determined using a BCA protein assay (Pierce, Rockford, IL, USA). For immunoprecipitation, Cells were lysed in RIPA buffer with protease inhibitors. Lysates were mixed with antibodies and protein A/G-beads UltraLink Resin (#35133, Pierce) and incubated at 4 °C overnight. Immunoprecipitates were washed five times in the lysis buffer before western blot analysis.

### Immunofluorescence analysis

After transfection with control siRNA or siTGase 2, CAKI-1 and ACHN cells were grown on coverslips and fixed in 4% paraformaldehyde for 10 min at room temperature. Fixed cells were stained with anti-p53 and anti-p62 antibodies. Alexa Fluor 488 (green) or Alexa Fluor 546 (red) conjugated secondary antibodies were used for visualization. DAPI was used to stain the nuclei (blue). Cells incubated with secondary antibodies alone were used as controls. Images were obtained using a Zeiss Axiovert 200M microscope.

### Preclinical xenograft tumor model

Xenografts were initiated in female BALB/c nude mice (6–8 weeks old; *n*=3/group). Briefly, ACHN-luciferase cells (4 × 10^5^) were inoculated subcutaneously near the scapulae. When tumors reached a volume of 100 mm^3^, oral treatment began with vehicle alone, GK921 (2 mg/kg/day),^3^ doxorubicin (1 mg/kg/day), or the drug combination, once per day, 5 days/week, for 35 days. The size of the primary tumors was measured every 2–3 days using calipers. Tumor volume (*V*; mm^3^) was calculated using the formula *V*=(*A* × *B*^2^)/2, where *A* is the longest diameter (mm) and *B* is the shortest diameter (mm). This study was reviewed and approved by the Institutional Animal Care and Use Committee (IACUC) of the National Cancer Center Research Institute, which is an Association for Assessment and Accreditation of Laboratory Animal Care International (AAALAC International) accredited facility that abides by the Institute of Laboratory Animal Resources guide (protocols: NCC-15-241).

### Imaging of tumor mass using bioluminescence

To monitor photon flux, mice were anesthetized with isoflurane inhalation, and 100 *μ*l of d-luciferin (7.5  mg/ml, Xenogen, PerkinElmer, Waltham, MA, USA) was intraperitoneally injected. Bioluminescence imaging with a CCD camera (IVIS, Xenogen) was initiated 5 min after the injection for 5 min, depending on the amount of luciferase activity. The data were expressed as photon flux (photons/s/cm^2^/steradian).

### Apoptosis assays

The Annexin V-FITC Apoptosis Detection kit (#556547, BD Pharmingen, San Diego, CA, USA) was used according to the manufacturer's instructions. Briefly, cells were harvested, washed twice with cold phosphate-buffered saline, and resuspended in 1 × binding buffer at a concentration of 1 × 10^6^ cells/ml. Cell suspension (100 *μ*l; 1 × 10^5^ cells) was transferred to a 5 ml culture tube and stained with 5 *μ*l of annexin V-FITC and 5 μl of propidium iodide in the dark for 15 min at room temperature. Then, 400 *μ*l of 1 × binding buffer was added to each tube. Data were acquired by flow cytometry within 1 h. TUNEL staining was performed on fixed cultured cells using the In situ Cell Death Detection Kit (Roche, Indianapolis, IN, USA).

### XTT assay

The inhibition of tumor cell growth caused by TGase 2 knockdown or TGase 2 inhibitor or doxorubicin or combination treatment with TGase 2 knockdown and doxorubicin or TGase 2 inhibitor and doxorubin was analyzed by XTT assay (Cell Proliferation assay Kit 30-1011 K, ATCC). Briefly, cells were plated at 2 × 10^4^ into a 96-well plate and incubated overnight to adhere. Cells were transfected with TG2 or control siRNA and then treated doxorubicin or GK921 as TG2 inhibitor 1 *μ*M for 8 h. The absorbance was measured at a wavelength of 475 nm using a Spectro star nano (BMG LABTECH, Ortenberg, Germany).

### Bioinformatics analysis of p62/*SQSTM1* and *TGM2* gene expression

To assess the mRNA expression levels of p62/*SQSTM1* and *TGM2* genes in clear cell RCC, we downloaded RNA-Seq data for kidney renal clear cell carcinoma (KIRC) samples from the Cancer Genome Atlas (TCGA).^29, 30^ We chose paired sequencing data, which consisted of matched cancer and normal tissues, and extracted the mRNA expression data, which were normalized by RNA-Seq expression estimation by expectation maximization,^30^ using a custom-made script. We drew box plots and calculated significance levels for the two genes using Student's *t*-test in R. For survival analyses, we obtained clinical information for the same KIRC patients^29–31^ and annotated the gene expression levels using our own scripts. Kaplan–Meier analyses were done using R software.^32^

### Statistical analysis

Statistical analysis was performed using the two-tailed Student's *t*-test for comparison between two groups. Significance was defined as *P*-value; *P<0.05, **P<0.01, ***P<0.001.

## Abbreviations

TGase 2: transglutaminase 2
RCC: renal cell carcinoma
HDM2: human double minute 2 homolog
p62: sequestosome 1
LC3: microtubule-associated protein 1 light chain 3 alpha

## References

[bib1] Folk JE. Transglutaminases. Annu Rev Biochem 1980; 49: 517–531. 6105840 10.1146/annurev.bi.49.070180.002505

[bib2] Ku BM, Kim DS, Kim KH, Yoo BC, Kim SH, Gong YD et al. Transglutaminase 2 inhibition found to induce p53 mediated apoptosis in renal cell carcinoma. FASEB J 2013; 27: 3487–3495. 23704086 10.1096/fj.12-224220

[bib3] Ku BM, Kim SJ, Kim N, Hong D, Choi YB, Lee SH et al. Transglutaminase 2 inhibitor abrogates renal cell carcinoma in xenograft models. J Cancer Res Clin Oncol 2014; 140: 757–767. 24610445 10.1007/s00432-014-1623-5 PMC11824106

[bib4] Oliner JD, Pietenpol JA, Thiagalingam S, Gyuris J, Kinzler KW, Vogelstein B. Oncoprotein MDM2 conceals the activation domain of tumour suppressor p53. Nature 1993; 362: 857–860. 8479525 10.1038/362857a0

[bib5] Haupt Y, Maya R, Kazaz A, Oren M. Mdm2 promotes the rapid degradation of p53. Nature 1997; 387: 296–299. 9153395 10.1038/387296a0

[bib6] Siliciano JD, Canman CE, Taya Y, Sakaguchi K, Appella E, Kastan MB. DNA damage induces phosphorylation of the amino terminus of p53. Genes Dev 1997; 11: 3471–3481. 9407038 10.1101/gad.11.24.3471 PMC316806

[bib7] Meek DW. Tumour suppression by p53: a role for the DNA damage response? Nat Rev Cancer 2009; 9: 714–723. 19730431 10.1038/nrc2716

[bib8] D'Eletto M, Farrace MG, Rossin F, Strappazzon F, Giacomo GD, Cecconi F et al. Type 2 transglutaminase is involved in the autophagy-dependent clearance of ubiquitinated proteins. Cell Death Differ 2012; 19: 1228–1238. 22322858 10.1038/cdd.2012.2 PMC3374086

[bib9] Kim PK, Hailey DW, Mullen RT, Lippincott-Schwartz J. Ubiquitin signals autophagic degradation of cytosolic proteins and peroxisomes. Proc Natl Acad Sci USA 2008; 105: 20567–20574. 19074260 10.1073/pnas.0810611105 PMC2602605

[bib10] Moscat J, Diaz-Meco MT, Albert A, Campuzano S. Cell signaling and function organized by PB1 domain interactions. Mol Cell 2006; 23: 631–640. 16949360 10.1016/j.molcel.2006.08.002

[bib11] Harper JW, Schulman BA. Structural complexity in ubiquitin recognition. Cell 2006; 124: 1133–1136. 16564007 10.1016/j.cell.2006.03.009

[bib12] Birgisdottir AB, Lamark T, Johansen T. The LIR motif - crucial for selective autophagy. J Cell Sci 2013; 126: 3237–3247. 23908376 10.1242/jcs.126128

[bib13] Efeyan A, Ortega-Molina A, Velasco-Miguel S, Herranz D, Vassilev LT, Serrano M. Induction of p53-dependent senescence by the MDM2 antagonist nutlin-3a in mouse cells of fibroblast origin. Cancer Res 2007; 67: 7350–7357. 17671205 10.1158/0008-5472.CAN-07-0200

[bib14] Bean LJ, Stark GR. Regulation of the accumulation and function of p53 by phosphorylation of two residues within the domain that binds to Mdm2. J Biol Chem 2002; 277: 1864–1871. 11707453 10.1074/jbc.M108881200

[bib15] Knippschild U, Milne D, Campbell L, Meek D. p53 N-terminus-targeted protein kinase activity is stimulated in response to wild type p53 and DNA damage. Oncogene 1996; 13: 1387–1393. 8875976

[bib16] Rodriguez MS, Desterro JM, Lain S, Lane DP, Hay RT. Multiple C-terminal lysine residues target p53 for ubiquitin-proteasome-mediated degradation. Mol Cell Biol 2000; 20: 8458–8467. 11046142 10.1128/mcb.20.22.8458-8467.2000 PMC102152

[bib17] Han BG, Cho JW, Cho YD, Jeong KC, Kim SY, Lee BI. Crystal structure of human transglutaminase 2 in complex with adenosine triphosphate. Int J Biol Macromol 2010; 47: 190–195. 20450932 10.1016/j.ijbiomac.2010.04.023

[bib18] Joerger AC, Fersht AR. Structure-function-rescue: the diverse nature of common p53 cancer mutants. Oncogene 2007; 26: 2226–2242. 17401432 10.1038/sj.onc.1210291

[bib19] LeMosy EK, Erickson HP, Beyer WFJr., Radek JT, Jeong JM, Murthy SN et al. Visualization of purified fibronectin-transglutaminase complexes. J Biol Chem 1992; 267: 7880–7885. 1348509

[bib20] Miyake M, Anai S, Fujimoto K, Ohnishi S, Kuwada M, Nakai Y et al. 5-fluorouracil enhances the antitumor effect of sorafenib and sunitinib in a xenograft model of human renal cell carcinoma. Oncol Lett 2012; 3: 1195–1202. 22783417 10.3892/ol.2012.662 PMC3392575

[bib21] Moscat J, Diaz-Meco MT. p62: a versatile multitasker takes on cancer. Trends Biochem Sci 2012; 37: 230–236. 22424619 10.1016/j.tibs.2012.02.008 PMC3531712

[bib22] Motzer RJ, Hutson TE, Tomczak P, Michaelson MD, Bukowski RM, Rixe O et al. Sunitinib versus interferon alfa in metastatic renal-cell carcinoma. N Engl J Med 2007; 356: 115–124. 17215529 10.1056/NEJMoa065044

[bib23] Rabinovitch RA, Zelefsky MJ, Gaynor JJ, Fuks Z. Patterns of failure following surgical resection of renal cell carcinoma: implications for adjuvant local and systemic therapy. J Clin Oncol 1994; 12: 206–212. 8270978 10.1200/JCO.1994.12.1.206

[bib24] Cheong H, Lu C, Lindsten T, Thompson CB. Therapeutic targets in cancer cell metabolism and autophagy. Nat Biotechnol 2012; 30: 671–678. 22781696 10.1038/nbt.2285 PMC3876738

[bib25] Guo JY, Xia B, White E. Autophagy-mediated tumor promotion. Cell 2013; 155: 1216–1219. 24315093 10.1016/j.cell.2013.11.019 PMC3987898

[bib26] Zheng B, Mao JH, Qian L, Zhu H, Gu DH, Pan XD et al. Pre-clinical evaluation of AZD-2014, a novel mTORC1/2 dual inhibitor, against renal cell carcinoma. Cancer Lett 2015; 357: 468–475. 25444920 10.1016/j.canlet.2014.11.012

[bib27] Ullen A, Farnebo M, Thyrell L, Mahmoudi S, Kharaziha P, Lennartsson L et al. Sorafenib induces apoptosis and autophagy in prostate cancer cells in vitro. Int J Oncol 2010; 37: 15–20. 20514392 10.3892/ijo_00000648

[bib28] Han JA, Park SC. Reduction of transglutaminase 2 expression is associated with an induction of drug sensitivity in the PC-14 human lung cancer cell line. J Cancer Res Clin Oncol 1999; 125: 89–95. 10190315 10.1007/s004320050247 PMC12199886

[bib29] Cancer Genome Atlas Research N. Comprehensive molecular characterization of clear cell renal cell carcinoma. Nature 2013; 499: 43–49. 23792563 10.1038/nature12222 PMC3771322

[bib30] Li B, Dewey CN. RSEM: accurate transcript quantification from RNA-Seq data with or without a reference genome. BMC Bioinformatics 2011; 12: 323. 21816040 10.1186/1471-2105-12-323 PMC3163565

[bib31] Cline MS, Craft B, Swatloski T, Goldman M, Ma S, Haussler D et al. Exploring TCGA Pan-Cancer data at the UCSC Cancer Genomics Browser. Sci Rep 2013; 3: 2652. 24084870 10.1038/srep02652 PMC3788369

[bib32] Kruithof CJ, Kooijman MN, van Duijn CM, Franco OH, de Jongste JC, Klaver CC et al. The Generation R Study: Biobank update 2015. Eur J Epidemiol 2014; 29: 911–927. 25527369 10.1007/s10654-014-9980-6

