# Supplementary material for: Renal cell carcinoma escapes death by p53 depletion through transglutaminase 2-chaperoned autophagy
Source: Cell Death Dis. 2016 Mar 31;7(3):e2163–. doi: 10.1038/cddis.2016.14 (PMC4823929; doi:10.1038/cddis.2016.14)
Supplement: Supplementary Figures [file cddis201614x1.ppt]

## Slide 1
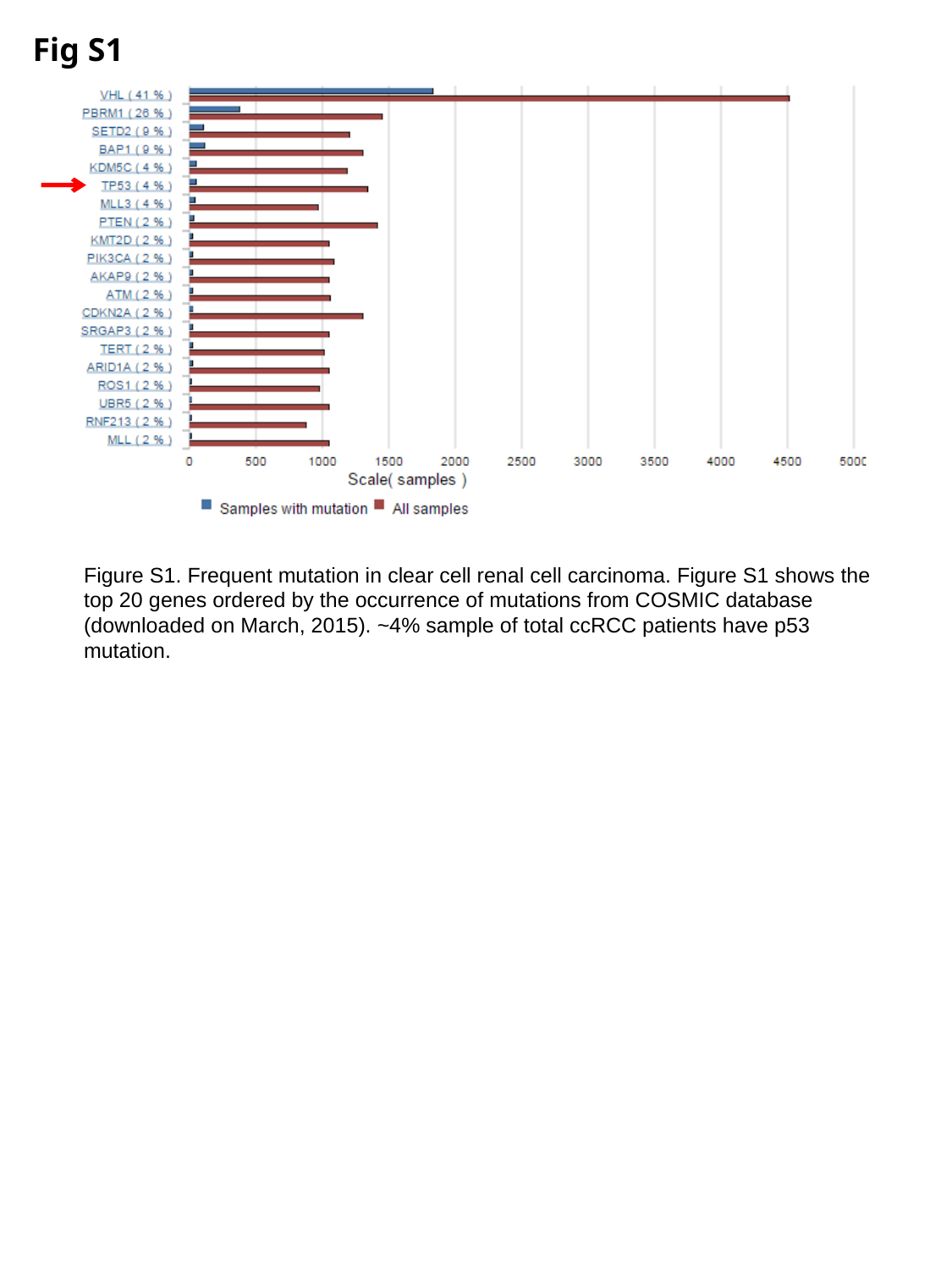

Fig S1
Figure S1. Frequent mutation in clear cell renal cell carcinoma. Figure S1 shows the top 20 genes ordered by the occurrence of mutations from COSMIC database (downloaded on March, 2015). ~4% sample of total ccRCC patients have p53 mutation.

## Slide 2
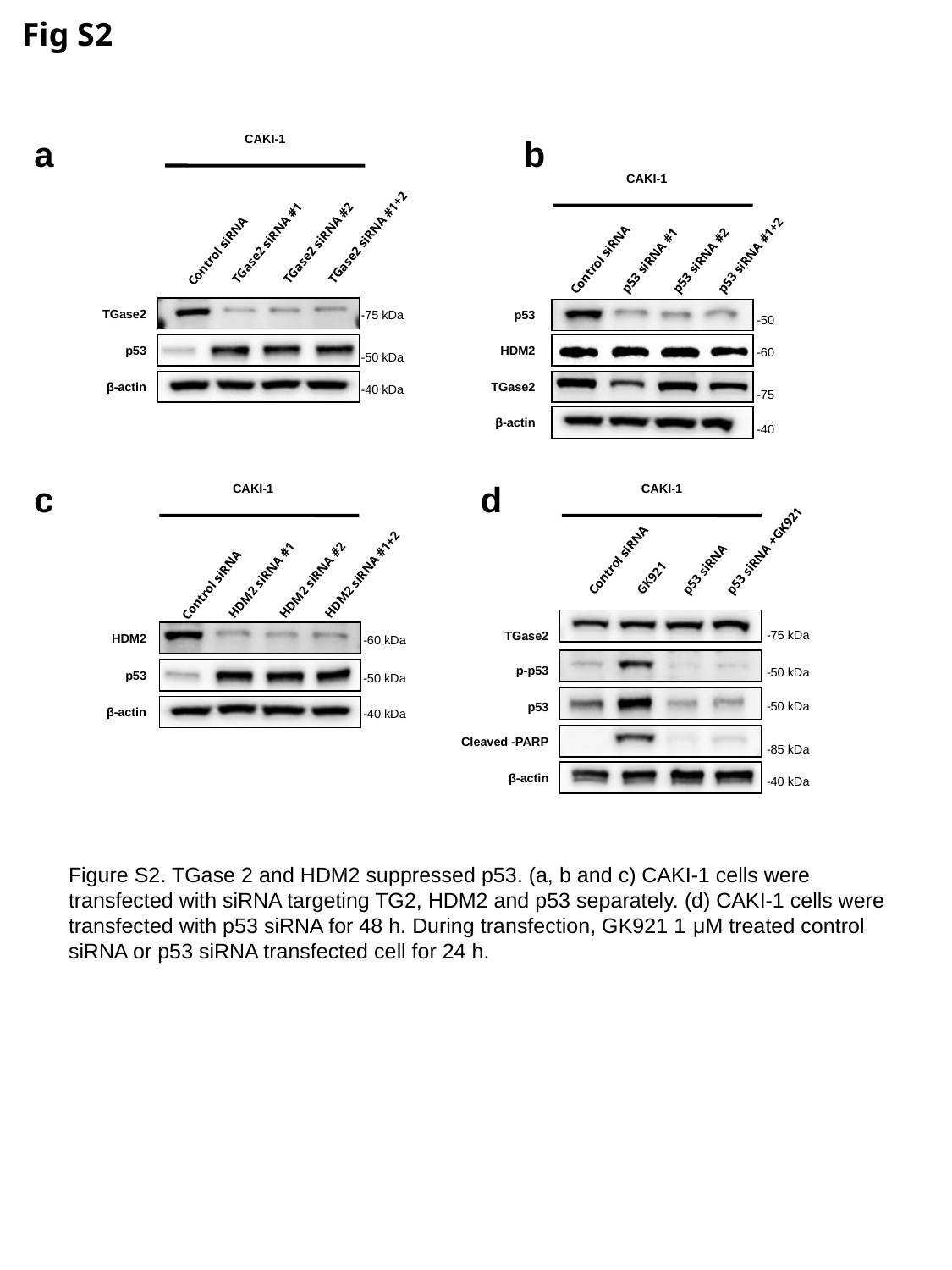

Fig S2
CAKI-1
a
b
CAKI-1
TGase2 siRNA #1+2
TGase2 siRNA #1
TGase2 siRNA #2
Control siRNA
p53 siRNA #1+2
Control siRNA
p53 siRNA #1
p53 siRNA #2
TGase2
-75 kDa
p53
-50
p53
HDM2
-60
-50 kDa
β-actin
TGase2
-40 kDa
-75
β-actin
-40
c
d
CAKI-1
CAKI-1
Control siRNA
GK921
p53 siRNA
p53 siRNA +GK921
HDM2 siRNA #1+2
HDM2 siRNA #1
HDM2 siRNA #2
Control siRNA
-75 kDa
TGase2
HDM2
-60 kDa
p-p53
-50 kDa
p53
-50 kDa
-50 kDa
p53
β-actin
-40 kDa
Cleaved -PARP
-85 kDa
β-actin
-40 kDa
Figure S2. TGase 2 and HDM2 suppressed p53. (a, b and c) CAKI-1 cells were transfected with siRNA targeting TG2, HDM2 and p53 separately. (d) CAKI-1 cells were transfected with p53 siRNA for 48 h. During transfection, GK921 1 μM treated control siRNA or p53 siRNA transfected cell for 24 h.

## Slide 3
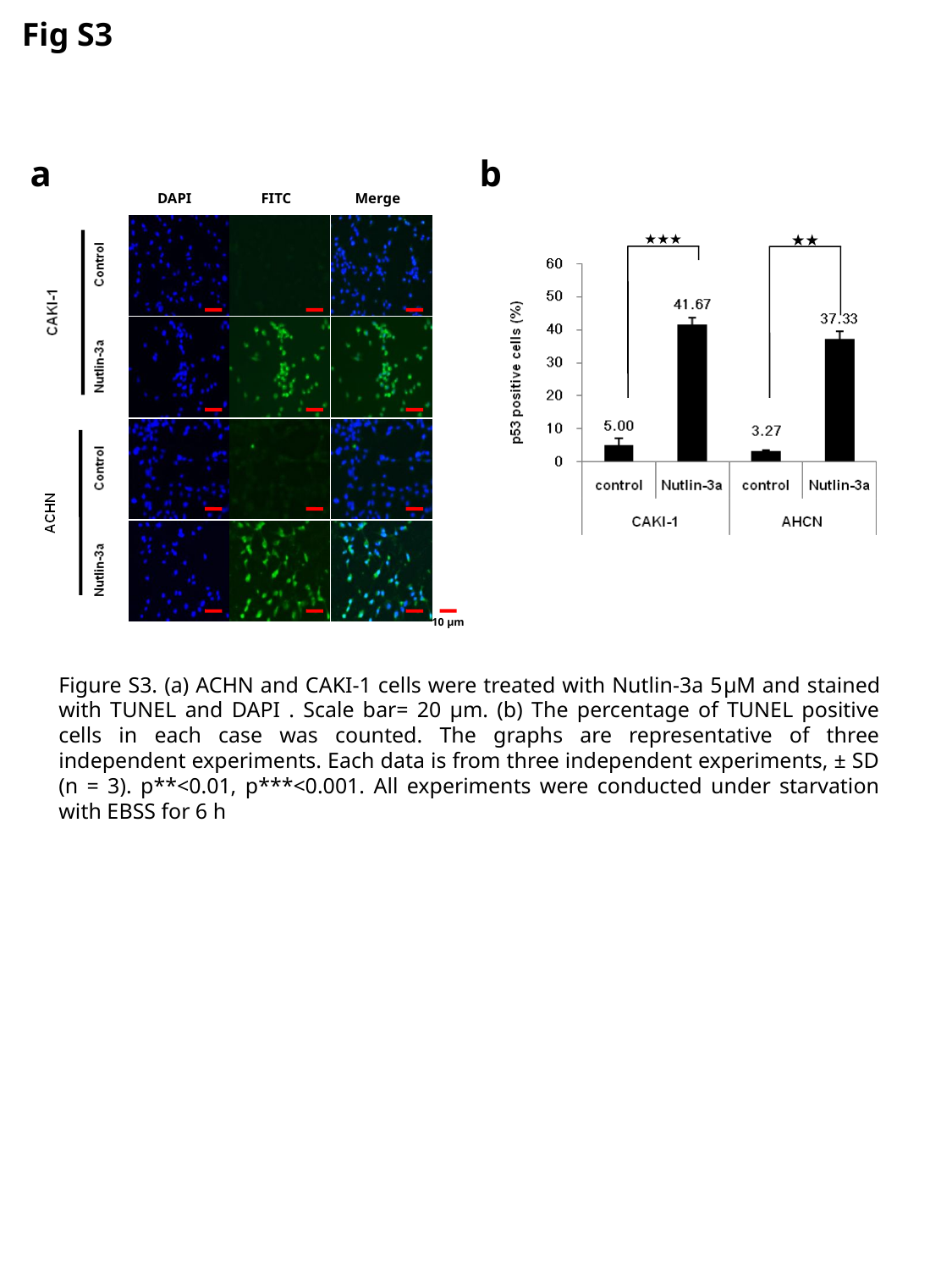

Fig S3
a
b
| DAPI | FITC | Merge |
| --- | --- | --- |
★★
★★★
10 μm
Figure S3. (a) ACHN and CAKI-1 cells were treated with Nutlin-3a 5μM and stained with TUNEL and DAPI . Scale bar= 20 μm. (b) The percentage of TUNEL positive cells in each case was counted. The graphs are representative of three independent experiments. Each data is from three independent experiments, ± SD (n = 3). p**<0.01, p***<0.001. All experiments were conducted under starvation with EBSS for 6 h
